# The impact of dynamic neuromuscular stabilization on postural control and balance in post-stroke patients with sarcopenia: A case report

**DOI:** 10.1097/MD.0000000000050639

**Published:** 2026-09-11

**Authors:** Jie Zhuang, Dorota Duhova, Lei Song, Juan Jin, Tingting Meng, Mengxue Sun, Zekai Hu, Yimeng Xu, Yimin Shen, Junwen Long, Jue Wang, Yueren Liu, Wenshu Guo, Jin Hu, Fen Rao, Lei Fang

**Affiliations:** a Department of Rehabilitation Medicine, The Second Rehabilitation Hospital Affiliated to Shanghai University of Traditional Chinese Medicine, Shanghai, China; b School of Rehabilitation Science, Shanghai University of Traditional Chinese Medicine, Shanghai, China; c School of Medicine, Shanghai University, Shanghai, China.

**Keywords:** balance, case report, dynamic neuromuscular stabilization, postural control, stroke rehabilitation

## Abstract

**Rationale:**

The coexistence of stroke and sarcopenia increases the risk of functional decline, falls, and disability. Dynamic Neuromuscular Stabilization (DNS) emphasizes diaphragmatic breathing, intra-abdominal pressure (IAP) regulation, and coordinated stabilizer activity; however, its role in post-stroke patients with sarcopenia remains uncertain.

**Patient concerns:**

The patient presented with significant balance impairment, a heightened fear of falling, and impaired coordinated movement of the right upper and lower limbs, resulting in substantial limitations in activities of daily living and reduced functional independence.

**Diagnoses:**

The patient met EWGSOP2 criteria for confirmed post-stroke sarcopenia.

**Interventions:**

The patient received a structured 5-week adjunctive DNS protocol (3 30-minute sessions per week) alongside conventional rehabilitation, progressing from supine diaphragmatic breathing and IAP generation through positional transitions (side-lying, prone, quadruped) to standing exercises. The protocol targeted diaphragmatic breathing, IAP generation, trunk stabilization, and movement dissociation across progressive developmental positions.

**Outcomes:**

Within-case pre-post changes were observed in balance (Berg Balance Scale: 35–42), trunk control (Trunk Impairment Scale: 17–20), functional mobility (Timed Up and Go: 47–28 seconds), non-hemiplegic grip strength (15–20 kg), and lower-limb functional strength (30-second Chair Stand Test: 6–11). The observed BBS, Trunk Impairment Scale, Timed Up and Go, grip-strength, and 30s-CST changes met or exceeded selected published minimal clinically important difference (MCID) estimates, but those estimates were derived from different stroke phases or other populations and are used only as contextual comparators, not as proof of clinical importance in chronic stroke with sarcopenia. The Sarcopenia and Quality of Life questionnaire change exceeded a published 7.35-point smallest detectable change, which indicates change beyond measurement error rather than an MCID.

**Lessons:**

This single-case report suggests that adjunctive DNS was feasible and well tolerated and was temporally associated with improved postural control and functional outcomes. These observations cannot establish a DNS-specific causal effect and should be tested in controlled studies.

## 1. Introduction

Stroke continues to impose a significant global burden as a leading cause of adult-acquired disability, with recent epidemiological data underscoring its persistent impact. According to the 2025 World Stroke Organization Global Stroke Fact Sheet, which incorporates the latest Global Burden of Disease 2021 estimates, stroke remains the world’s second leading cause of death and a primary contributor to disability-adjusted life-years lost, with approximately 11.9 million new cases and over 93.8 million survivors annually.^[1,2]^ Its strong association with advanced age places survivors at heightened risk for developing sarcopenia, a progressive syndrome characterized by loss of skeletal muscle mass, strength, and function. Further research indicates that pre-stroke or post-stroke sarcopenia can increase the risk of poor functional outcomes by approximately 2.71-fold and is associated with significantly reduced community discharge rates, thereby substantially exacerbating the dual burden on both individuals and the healthcare system.^[3]^ Epidemiological studies report a disproportionately high prevalence of sarcopenia in stroke survivors, estimated between 14% and 54%.^[4]^ A recent systematic review and meta-analysis reported a pooled overall prevalence of stroke-related sarcopenia of 42%. The review further highlighted that prevalence estimates varied according to the diagnostic criteria applied (e.g., approximately 50.4% using Asian Working Group for Sarcopenia criteria vs 45.5% using European Working Group on Sarcopenia in Older People (EWGSOP) criteria) and were significantly higher in the early post-stroke phase (<1 month, ~50%) compared to the chronic phase (≥6 months, ~34%).^[5]^ These findings underscore the high and stage-dependent burden of post-stroke sarcopenia and emphasize the importance of employing consistent and specific diagnostic criteria, such as EWGSOP2,^[6]^ for accurate assessment in both clinical and research settings. Postural instability and balance deficits represent highly prevalent and debilitating post-stroke sequelae, substantially increasing fall risk and limiting functional independence.^[7,8]^ The convergence of neurological insult and sarcopenia synergistically exacerbates functional decline and frailty. Stroke induces rapid deleterious skeletal muscle alterations that directly map onto the diagnostic triad of sarcopenia as defined by the EWGSOP2 criteria: low muscle strength, low muscle quantity or quality, and low physical performance. These alterations manifest as bilateral weakness (including detectable deficits in the non-paretic limb within one week), substantial loss of muscle mass (approximately 24% in paretic limbs) accompanied by qualitative degradation such as fat infiltration, and consequently, severely impaired physical performance in gait, balance, and endurance (see Table 1).^[9]^ Contributing factors encompass profound physical inactivity during acute care, often amounting to <40 minutes daily; malnutrition affecting approximately 49% of survivors; dysphagia; spasticity; and unique neurogenic changes distinct from simple disuse atrophy.^[10,11]^ Sarcopenia following stroke is strongly associated with severe adverse outcomes. Its presence is associated with poorer functional recovery, significantly reduced likelihood of community discharge, prolonged rehabilitation stays, and lower discharge motor functional independence measure scores.^[12]^ Core stability is also increasingly recognized as relevant to coordinated movement during stroke rehabilitation.^[13]^

**Table 1:** Correspondence between post-stroke muscle alterations and EWGSOP2 diagnostic domains for sarcopenia.

Post-stroke alteration	Pathophysiological mechanism	Corresponding EWGSOP2 domain
Bilateral limb weakness (paretic & non-paretic)	Neurological insult, disuse, neurogenic changes	Low muscle strength
Significant reduction in muscle mass, fat infiltration, architectural disruption	Neurogenic atrophy, catabolic state, immobility; altered muscle composition, myosteatosis	Low muscle quantity or quality
Impaired gait speed, balance, endurance	Combined effect of weakness, poor trunk control, deconditioning	Low physical performance

EWGSOP = European Working Group on Sarcopenia in Older People.

Effective postural control fundamentally relies upon robust core stability generated through the integrated function of deep trunk musculature, including the diaphragm, pelvic floor, and transversus abdominis, which modulates intra-abdominal pressure (IAP).^[14]^ Stroke disrupts neurological control of this system, while post-stroke sarcopenia directly weakens its constituent muscles. Diaphragmatic dysfunction and consequent IAP dysregulation represent key pathophysiological mechanisms underpinning postural instability in this population.^[15,16]^ Conventional rehabilitation approaches such as proprioceptive neuromuscular facilitation or the Bobath concept have a well-established history in stroke rehabilitation and demonstrate efficacy in addressing spasticity and facilitating movement patterns.^[17–19]^ However, they often underemphasize the critical roles of diaphragmatic function and IAP regulation in core stability, which are fundamental for postural control. Furthermore, these approaches may not fully integrate or adapt to the specific physiological constraints imposed by concomitant sarcopenia, such as generalized muscle weakness and altered neuromuscular efficiency.

This therapeutic gap underscores the potential relevance of Dynamic Neuromuscular Stabilization (DNS) – an approach that reactivates innate developmental motor patterns emphasizing diaphragmatic breathing, IAP optimization, and integrated stabilizer synergy – for patients with post-stroke sarcopenia. DNS is grounded in ontogenetic developmental kinesiology and activates deep stabilizing muscles through systematic progression across developmental positions (such as supine, side-lying, prone, quadruped, and standing). Key muscles engaged include the diaphragm, transversus abdominis, pelvic floor muscles, multifidus, and the deep cervical flexors, which together regulate IAP and establish proximal stability for distal mobility. DNS principles have been shown to increase abdominal-wall tension across postures, whereas observational stroke research has associated low appendicular muscle mass with poorer functional outcomes.^[20,21]^ However, these studies do not establish the effectiveness of DNS in chronic post-stroke sarcopenia. We therefore hypothesized that a structured DNS protocol, by targeting the compromised core stability and neuromuscular inefficiency common to both conditions, could offer a relevant adjunctive intervention. Given the paucity of robust evidence supporting DNS specifically within this complex subgroup, we implemented a targeted DNS protocol adjunctive to standard care in a chronic stroke patient with significant sarcopenia to describe changes in postural control and balance function.

## 2. Case description

This report describes a 69-year-old man who presented with substantial functional limitations 5 months after a conservatively managed left thalamic hemorrhage. His functional status was characterized by marked balance impairment and a heightened fear of falling, resulting in substantial restrictions in activities of daily living and reduced functional independence. He also had a history of prolonged post-stroke immobility. A comprehensive neurological examination was performed at baseline, including assessments of consciousness and the motor and sensory systems. The patient was alert and oriented. Cranial nerve examination revealed mild right-sided facial weakness (CN VII) and mild dysarthria, but no visual field deficits, diplopia, or dysphagia. Motor examination confirmed right hemiparesis with Medical Research Council grades: right shoulder abduction 3/5, elbow flexion 4/5, wrist extension 2/5, hip flexion 3/5, knee extension 4/5, and ankle dorsiflexion 2/5. Left-sided strength was within normal limits (5/5 throughout). Muscle tone was increased on the right side (Modified Ashworth Scale 1+ in elbow flexors and plantar flexors). Sensation to light touch and proprioception was diminished in the right upper and lower limbs. Sarcopenia was classified using EWGSOP2 because this framework prioritizes low muscle strength and confirms sarcopenia with low muscle quantity or quality. The patient had low non-hemiplegic (left) handgrip strength (15 kg; <27 kg for men) and a low appendicular skeletal muscle mass index (6.2 kg/m^2^; <7.0 kg/m^2^ for men), confirming sarcopenia under EWGSOP2. Bioelectrical Impedance Analysis was performed with an InBody S10 in the morning after an overnight fast; the patient voided immediately before measurement, avoided exercise and alcohol for 24 hours and food for at least 8 hours, avoided fluid intake for 2 hours, rested supine for 5 minutes, and had the 8 tactile electrodes placed bilaterally on the thumbs, middle fingers, heels, and forefeet according to the manufacturer’s procedure. The 4-meter usual gait speed was 0.5 m/s and was used to describe physical performance rather than to confirm the diagnosis. As an external sensitivity classification relevant to an Asian/Chinese patient, the Asian Working Group for Sarcopenia 2019 thresholds also identified low muscle mass (appendicular skeletal muscle mass index < 7.0 kg/m^2^) and low muscle strength (handgrip < 28 kg).^[22,23]^ In stroke, however, neither non-hemiplegic handgrip strength nor gait speed is a stroke-specific validated diagnostic standard: severe hemiparesis can produce floor effects in the paretic hand and gait assessment, while non-hemiplegic handgrip is a pragmatic substitute. These limitations should temper interpretation of the diagnostic classification. The patient’s baseline demographic and clinical characteristics and assessment results are summarized in Table 2. The patient was subsequently transferred to our institution for continued rehabilitation. Prior to this transfer, he had been undergoing consistent rehabilitation therapy (including proprioceptive neuromuscular facilitation/Bobath-based limb training, gait training, and strength exercises) at another facility, but satisfactory functional recovery had not been achieved. Our evaluation identified significant synergistic movement patterns in both the right upper and lower extremities. Postural assessment revealed pronounced rib flare. During the DNS upper-extremity elevation test, a loss of IAP was observed through palpation of lateral abdominal-wall tension and visual assessment of abdominal-wall configuration, a standard clinical assessment method within the DNS framework.^[24]^ His respiratory pattern was predominantly thoracic, indicating compromised diaphragmatic function. These impairments contributed to difficulty during ambulation, with increased muscular co-contraction, reduced movement fluidity, excessive trunk rotation during right lower-extremity advancement, and a persistently flexed, spastic posture of the right upper limb. Consequently, an individualized rehabilitation regimen was designed, incorporating DNS-based exercises and postural-correction strategies to address these deficits.

**Table 2:** Baseline information.

Project	Content	Project	Content
Age	69	BBS	35
Gender	Male	TIS	17
Type of stroke	Intracerebral hemorrhage	TUG (s)	47
Duration (mo)	5	GS (kg)	15 < 27
Hemiplegic side	Right	30s-CST (s)	6
Heigh (cm)	165	ASMI (kg/m^2^)	6.2 < 7.0
Weight (kg)	75	Gait speed (m/s)	0.5 ≤ 0.8
BMI (kg/m^2^)	27.5	FMA	70
Underlying condition	Hypertension	SarQoL	55

30s-CST = 30-second Chair Stand Test, ASMI = appendicular skeletal muscle mass index, BBS = Berg Balance Scale, BMI = body mass index, FMA = Fugl-Meyer assessment scale, GS = grip strength, SarQoL = Sarcopenia and Quality of Life, TIS = Trunk Impairment Scale, TUG = Timed Up and Go.

The patient had a history of hypertension. Before the index stroke, he had been prescribed antihypertensive medication but reported irregular adherence. After the stroke, he resumed antihypertensive treatment and reported taking the prescribed medication regularly during the rehabilitation period.

The Ethics Committee of the Second Rehabilitation Hospital of Shanghai approved this case report (No. 2025-12-01), and written informed consent for publication was obtained from the patient.

**Figure 1.**
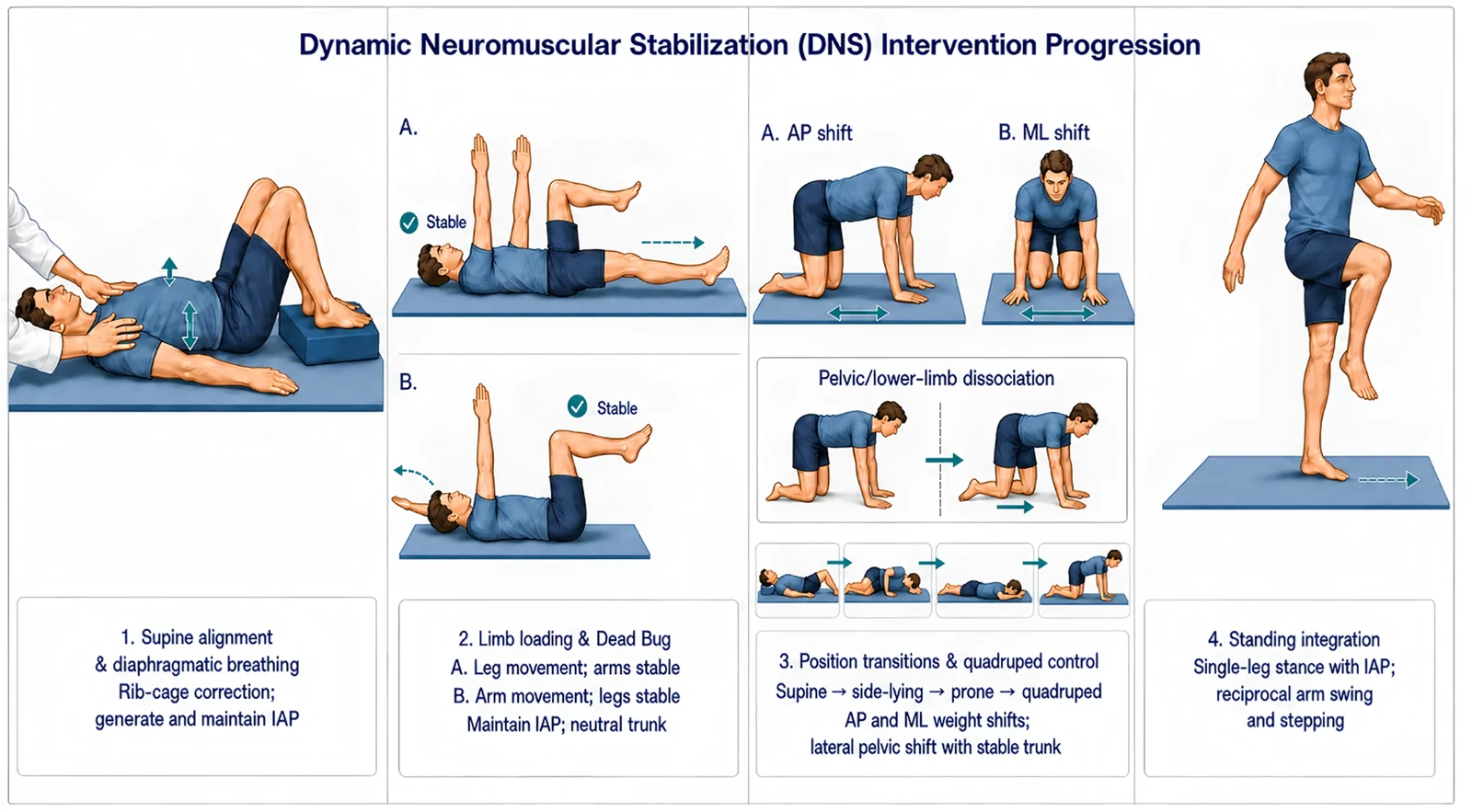
Staged DNS intervention. The intervention progressed from (1) supine alignment and diaphragmatic breathing, emphasizing rib-cage correction and the generation and maintenance of IAP, through (2) limb loading and dead-bug exercises while maintaining trunk neutrality and IAP, to (3) position transitions and quadruped control with anteroposterior (AP) and mediolateral (ML) weight shifts and pelvic–lower-limb dissociation. The final phase (4) integrated standing control through single-leg stance with IAP, reciprocal arm swing, and stepping. DNS = dynamic neuromuscular stabilization, IAP = intra-abdominal pressure.

The rehabilitation protocol commenced with the patient positioned supine, where passive postural correction was performed to achieve optimal alignment of the head, trunk, and pelvis. Manual compression was applied bilaterally to the ribcage in an inferior and caudal direction to passively address rib flare. This facilitated improved conditions for diaphragmatic descent. Within this corrected alignment, the patient was instructed in diaphragmatic breathing exercises to promote the generation of IAP, aiming for a cylindrical abdominal configuration. Once consistent IAP generation was established, progressive limb loading was introduced; the patient practiced maintaining IAP while actively elevating either the upper or lower limbs. Training then advanced to the Dead Bug position, focusing on segmental movement dissociation: lower limbs performed controlled alternating movements while the upper limbs remained stable, and conversely, upper limbs moved while lower limbs were stabilized. This exercise targeted trunk stability and movement dissociation. After adequate sagittal-plane stability had been achieved, the patient practiced controlled positional transitions, moving sequentially from supine to side-lying, prone, and finally the quadruped position. Scapular stability, pelvic stability, and overall alignment were closely monitored during transitions, with manual corrections provided immediately if instability or malalignment occurred. While in the quadruped position, exercises included anterior-posterior and medio-lateral weight shifting, along with pelvic and lower extremity dissociation drills requiring lateral shifting of the pelvis and lower limbs while maintaining upper trunk stability. Ultimately, upon demonstrating competent postural control across these positions, training progressed to standing. This final stage involved maintaining IAP during single-leg stance and practicing coordinated upper limb swing with stepping movements. Throughout standing exercises, meticulous attention was paid to minimizing compensatory trunk rotation. For reproducibility, the positions, exercise content, dosage parameters, and progression criteria are detailed in Supplementary Table S1, Supplemental Digital Content 1. The staged intervention sequence is illustrated in Figure 1.

A structured safety monitoring protocol was implemented throughout the intervention. Adverse events (AEs) were systematically recorded after each session using a standardized form aligned with the Consolidated Standards of Reporting Trials (CONSORT) harms recommendations. Monitored parameters included pain, excessive fatigue, dizziness, and other potential discomforts. Any moderate or severe AE would have prompted session termination and clinical review. Serious AEs, as defined by standard regulatory criteria (e.g., hospitalization, significant disability), would have led to study discontinuation and ethics reporting. Patient tolerance was routinely assessed. No AEs or serious AEs occurred, indicating that the protocol was well tolerated in this patient.

The protocol was implemented over 5 weeks, with 3 30-minute sessions per week, alongside the patient’s conventional rehabilitation programme. A session record was completed after each intervention session to document completion, adverse reactions, and patient acceptability. Assessments were conducted immediately before the intervention began and immediately after the five-week intervention concluded. All assessments were performed by a physiotherapist blinded to the intervention protocol; no interim assessments were conducted to avoid reactivity and order effects. Because this was a single pre-post case without repeated baseline observations or an estimate of within-person variability, inferential statistical testing and standardized effect-size calculation were not considered appropriate. Results were therefore summarized descriptively using raw change scores and, where informative, percentage changes. BBS increased from 35 to 42 (+7 points; +20.0%), Trunk Impairment Scale (TIS) from 17 to 20 (+3 points; +17.6%), Timed Up and Go (TUG) decreased from 47 to 28 seconds (−19 seconds; −40.4%), non-hemiplegic grip strength increased from 15 to 20 kg (+5 kg; +33.3%), and 30-second Chair Stand Test (30s-CST) repetitions increased from 6 to 11 (+5; +83.3%). Against the cited thresholds, the 7-point BBS increase exceeded the 5-point minimal clinically important difference (MCID) reported for assisted walkers with early subacute stroke; the same study reported 4 points in unassisted walkers. The 3-point TIS increase equaled the 3-point MCID reported in acute stroke. The 19-second TUG reduction exceeded the 8-second MCID cited in a chronic-stroke feasibility study. The 5-kg grip-strength increase equaled the 5.0-kg MCID reported for the dominant side after stroke, and the 5-repetition 30s-CST increase exceeded the 2-repetition MCID used in community-dwelling older adults.^[25–29]^ These comparisons do not validate any threshold for this patient or establish clinical importance in chronic stroke with sarcopenia. Sarcopenia and Quality of Life questionnaire (SarQoL) increased from 55 to 80 (+25 points). In sarcopenic adults, the SarQoL overall score has an individual smallest detectable change (SDC) of 7.35 points; the present change exceeds that measurement-error threshold, but SDC is not an MCID and does not establish clinical importance in chronic stroke with sarcopenia.^[30]^ These results are summarized in Table 3.

**Table 3:** Comparisons of change in BBS, TIS, TUG, GS, and 30s-CST.

Assessment	Pretest	Post-test
BBS	35	42
TIS	17	20
TUG (s)	47	28
GS (kg)	15	20
30s-CST (s)	6	11
SarQoL	55	80

30s-CST = 30-second Chair Stand Test, BBS = Berg Balance Scale, GS = grip strength, SarQoL = Sarcopenia and Quality of Life, TIS = Trunk Impairment Scale, TUG = Timed Up and Go.

Treatment fidelity was supported by a session record documenting completion of the prescribed intervention, adverse reactions, and patient acceptability. The assessor was a physiotherapist blinded to the intervention protocol; blinding of the treating therapist and participant was not possible in this rehabilitation case report.

The applicability of these comparisons is limited. Published BBS minimal important change estimates in stroke range from approximately 3 to 6 points and vary with stroke phase, severity, walking-assistance status, and the selected anchor. An anchor-based MID of >2 points (approximately 3 integer points) was reported in chronic stroke, whereas a MIC of ≥6 points was reported early after stroke.^[31,32]^ The 5-point value was retained because it was the estimate for assisted walkers in the cited early-subacute cohort, not because it is universal. The TIS 3-point MCID was established in acute stroke and has not been validated in chronic stroke with sarcopenia. The TUG 8-second value was retained as the estimate cited in the chronic-stroke source, not because it is a preferred universal threshold. Published TUG change thresholds reported across stroke studies span approximately 3.8 to 8.5 seconds, reflecting differences in stroke phase, severity, study design, and analytical method; therefore, 8 seconds is used only as a contextual comparator rather than a fixed cutoff.^[27,33]^ The grip-strength 5.0-kg value was reported for the dominant side after stroke, whereas this case used the non-hemiplegic left hand as a pragmatic measure; dominance and non-hemiplegic status are not interchangeable. The 30s-CST 2-repetition value was used in independent community-dwelling older adults and has not been validated in stroke with sarcopenia. No SarQoL MCID validated for chronic stroke with sarcopenia was available; the reported 7.35-point SDC is a measurement-error threshold, not a clinical-importance threshold. Accordingly, meeting or exceeding these values provides descriptive context only and does not establish a DNS-specific effect or clinically important individual change in this case.

Furthermore, the Balance Manager system (NeuroCom, USA) was used to assess limits of stability in 4 directions. Parameters included maximal excursion (MXE), directional control, and movement velocity. The raw pre-post values showed improvement in selected lateral and posterior weight-shifting measures (Table 4); these descriptive observations do not isolate the effect of DNS.

**Table 4:** Comparison of change in Balance Manager system.

Front	Pretest	Post-test
MXE (%)	30	28
DCL (%)	79	75
MVL (degrees/s)	1.1	1.0
Back		
MXE (%)	34	69
DCL (%)	50	72
MVL (degrees/s)	0.8	1.2
Right		
MXE (%)	38	58
DCL (%)	78	79
MVL (degrees/s)	0.9	1.2
Left		
MXE (%)	44	45
DCL (%)	70	85
MVL (degrees/s)	1.8	2.0

DCL = directional control, MVL = movement velocity, MXE = maximal excursion.

In addition, process records indicated excellent intervention adherence and tolerability: all 15 planned DNS sessions were completed (100% completion rate) with no AEs reported. The patient reported that the exercises felt “gradually more natural” and noted reduced anxiety during standing and walking tasks as the programme progressed. These process measures further support the feasibility and acceptability of DNS in this complex clinical presentation.

## 3. Discussion

This case report describes within-case changes observed after a structured 5-week adjunctive DNS programme in a 69-year-old man with chronic post-stroke impairments and sarcopenia. The report is intended to describe feasibility, tolerability, and hypothesis-generating clinical observations rather than to demonstrate therapeutic efficacy.

Between baseline and the end of the five-week intervention, BBS increased from 35 to 42, TIS from 17 to 20, TUG decreased from 47 to 28 seconds, non-hemiplegic grip strength increased from 15 to 20 kg, and 30s-CST performance increased from 6 to 11 repetitions. SarQoL increased from 55 to 80, and the Balance Manager assessment showed improvement in selected posterior and lateral limits-of-stability measures. The BBS, TIS, TUG, grip-strength, and 30s-CST changes met or exceeded selected published MCID estimates, and the SarQoL change exceeded a published SDC. These pre-post observations remain descriptive because the thresholds were derived from non-comparable populations; they cannot be interpreted as proof of a treatment effect or of clinically important change in chronic stroke with sarcopenia.

**Figure 2.**
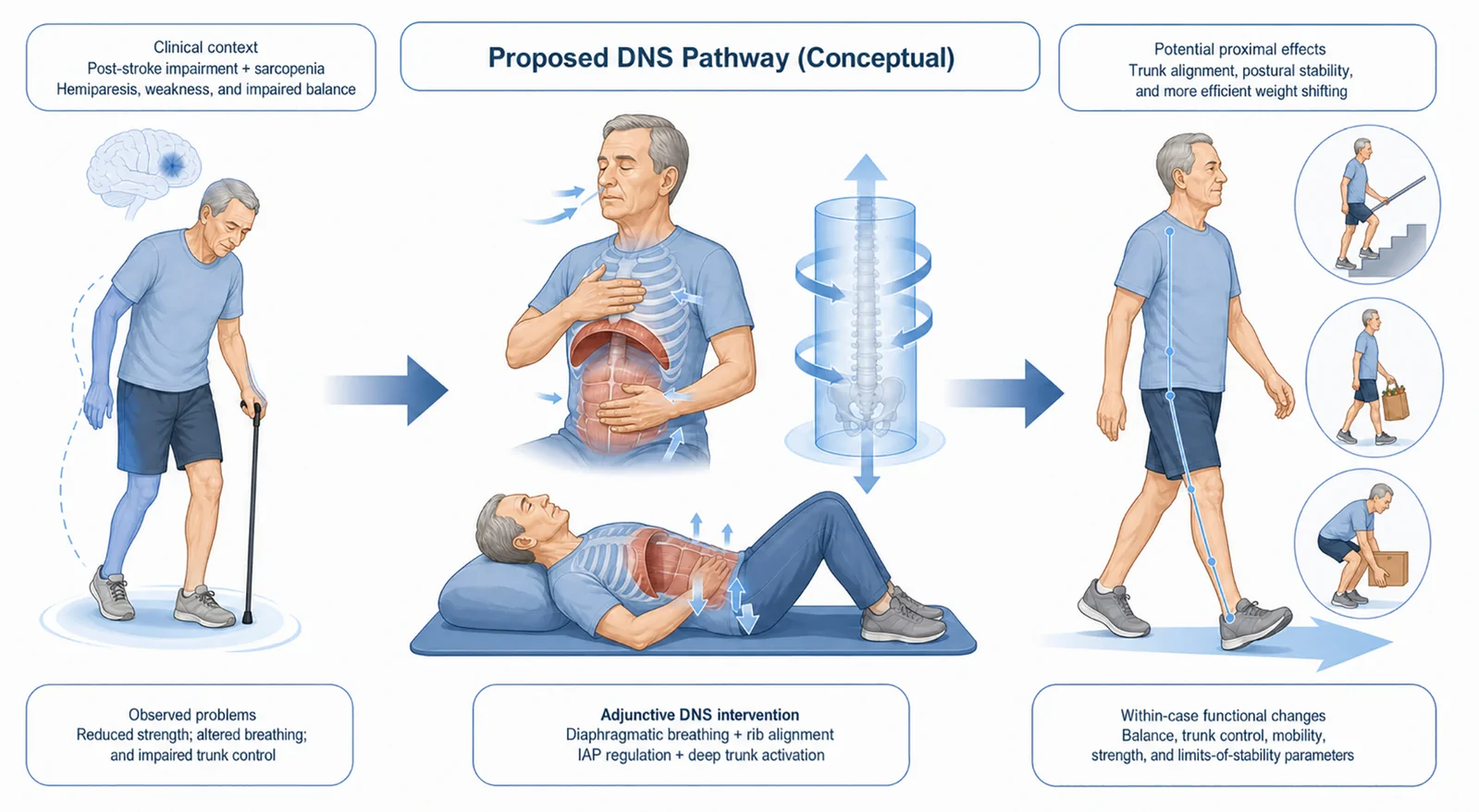
Conceptual framework linking DNS training to the observed functional changes in this case. Post-stroke motor impairment, sarcopenia, weakness, altered breathing, and impaired trunk control provided the clinical context. Adjunctive DNS emphasized diaphragmatic breathing, rib alignment, IAP regulation, and deep-trunk activation. These mechanisms may support trunk alignment, postural stability, and weight shifting, potentially contributing to within-case changes in balance, trunk control, mobility, strength, and limits-of-stability parameters. This is a conceptual hypothesis only; the proposed pathway was not directly measured and does not establish causality. DNS = dynamic neuromuscular stabilization, IAP = intra-abdominal pressure.

DNS is hypothesized to support postural control by encouraging diaphragmatic excursion, IAP regulation, coordinated activation of deep trunk stabilizers, and movement dissociation. Evidence from related DNS or core-stabilization studies provides biological plausibility for this framework.^[34]^ However, it does not establish the proposed pathway in the present patient. In this case, diaphragm imaging and electromyography were not obtained. Figure 2 therefore presents a proposed mechanism, not a demonstrated causal pathway. The staged progression from alignment and breathing to limb loading, transitions, quadruped weight shifting, and standing was selected to provide a structured rehabilitation stimulus; its relative contribution cannot be separated from concurrent conventional rehabilitation or other time-related factors.^[35,36]^

The coexistence of post-stroke motor impairment and sarcopenia provides a rationale for studying interventions that integrate trunk control, respiratory-stabilizer function, and task-oriented mobility. In the present patient, improvements in balance, strength, and self-reported quality of life occurred during a period that included both DNS and conventional rehabilitation; the contribution of each component is unknown. Previous stroke-rehabilitation literature on motor relearning, core exercise, and trunk training reports heterogeneous or modest balance effects.^[37–39]^ These findings reinforce the need for controlled studies rather than supporting superiority of DNS over established rehabilitation approaches.

Methodological limitations should be acknowledged. First, the single-case design precludes causal inference and limits generalizability. The observed changes may reflect natural recovery, concurrent conventional rehabilitation, expectancy effects, or measurement variability, and their sustainability is unknown without long-term follow-up. Because there were no repeated baseline observations or an estimate of within-person variability, inferential statistical testing and standardized effect-size calculation were not appropriate. Second, although the outcome assessor was blinded to the protocol, the treating therapist and participant could not be blinded. Third, no diaphragm ultrasonography, electromyography, or other direct physiological measure was obtained to test the proposed mechanism. Finally, no stroke-specific validated diagnostic standard was available. Non-hemiplegic handgrip strength was used pragmatically because paretic-side grip assessment was unreliable, and gait speed may be constrained by hemiparesis; Bioelectrical Impedance Analysis results can also vary with hydration despite the standardized procedure. Outcome MCIDs are also population-, stroke-phase-, severity-, side-, and anchor-dependent, and none has been validated specifically for chronic stroke with sarcopenia. This uncertainty is particularly relevant to the heterogeneous TUG estimates, use of a dominant-side grip-strength threshold for a non-hemiplegic-side measurement, and the community-derived 30s-CST threshold. The SarQoL comparator is an SDC rather than an MCID. Therefore, meeting or exceeding these thresholds cannot establish clinically important individual change or a DNS-specific effect.

Future research should adopt a structured agenda that progresses logically from mechanistic validation to definitive clinical trials. Initial studies must incorporate direct physiological measures, such as diaphragm ultrasonography and electromyography of deep trunk stabilizers, to confirm the proposed neuromuscular mechanisms of DNS in this population. Building on this foundation, the priority should shift to conducting multicenter randomized controlled trials comparing adjunctive DNS against conventional rehabilitation in post-stroke patients with sarcopenia, employing standardized protocols and instrumented functional outcomes. These trials should also explore optimal intervention parameters and potential protocol augmentations to address residual deficits like anterior weight-shifting limitation. Finally, translational studies investigating long-term efficacy, cost-effectiveness, and implementation in diverse clinical settings are essential to determine the sustainable role of DNS in managing this complex comorbidity. This cohesive research pathway directly addresses the key gaps – lack of mechanistic evidence and uncertain long-term impact – highlighted by the present case report.

Clinically, this report identifies features that may justify future study of DNS in post-stroke sarcopenia, including rib flare, thoracic-breathing predominance, apparent loss of IAP during limb elevation, and marked synergistic interference during functional movement. These observations should not be used as treatment-selection criteria until they are prospectively evaluated. Wider implementation would also require appropriately trained therapists and formal assessment of feasibility, safety, and cost-effectiveness across clinical settings.

This case provides a descriptive signal that a core-focused DNS program can be delivered alongside conventional rehabilitation in a patient with chronic stroke and sarcopenia. It does not demonstrate that DNS caused the observed changes or that it should replace established rehabilitation. Prospective controlled studies with direct mechanistic measures and long-term follow-up are required before clinical recommendations can be made.

## 4. Conclusion

This case report describes improvements in postural control, balance, functional mobility, and strength measures during a five-week period of adjunctive DNS and conventional rehabilitation in a patient with post-stroke sarcopenia. DNS was feasible and well tolerated in this case; however, the design cannot attribute the observed changes specifically to DNS.

The proposed links among diaphragmatic function, IAP regulation, trunk stability, and functional performance remain hypotheses because direct physiological measures were not obtained. Controlled studies are needed to test mechanisms, compare DNS with conventional rehabilitation, define appropriate candidates, and assess long-term outcomes.

## 5. Patient perspective

### 5.1. Challenges before the intervention

Five months after my stroke, I faced severe daily struggles. Walking was marked by profound instability, and my right arm and leg moved in a stiff, locked pattern, which filled me with constant anxiety about falling. This fear was debilitating and turned simple tasks into sources of stress, also placing a significant care burden on my family. My world felt very limited.

### 5.2. Functional and psychological changes after DNS training

Participating in the DNS training marked a turning point. I gradually gained much better trunk control and learned to separate the movements of my upper and lower limbs. This newfound control translated directly into daily life: I can now shift my weight more smoothly while standing, maintain a steadier posture while sitting, and perform activities like putting on shoes, washing, or picking items off the floor with greater safety and coordination. The improvement in my balance and strength meant I could get up from a chair much more quickly and confidently. I have progressed to walking short distances indoors without a cane, using it only for longer walks as a precaution. Most importantly, I regained independence in essential self-care tasks like dressing and transferring from sitting to standing. This tangible functional progress has significantly reduced my anxiety. Moving from constant fear to being able to manage daily activities alone has greatly restored my confidence in navigating life.

### 5.3. Ongoing confidence and future outlook

The reduction in fear and increase in independence have been as valuable as the physical improvements. Needing less help from my wife has lightened the load on both of us. While I know continued practice is important, the gains from this program have given me a hopeful and more positive outlook on my recovery journey. I feel reengaged in my own life.

## Acknowledgments

We sincerely thank the patient and his family for their cooperation and support and the rehabilitation team for their contributions to the intervention and assessment. We also gratefully acknowledge the financial support provided by the National Natural Science Foundation of China (Grant No. 82274671) and the Research Project of the Second Rehabilitation Hospital of Shanghai (No. Y2025-07).

## Author contributions

**Conceptualization:** Mengxue Sun, Yueren Liu, Wenshu Guo.

**Formal analysis:** Lei Song, Juan Jin, Tingting Meng.

**Investigation:** Jie Zhuang, Dorota Duhova.

**Methodology:** Zekai Hu, Yimeng Xu, Yimin Shen, Junwen Long, Jue Wang, Jin Hu, Fen Rao.

**Supervision:** Lei Fang.

**Writing – original draft:** Jie Zhuang, Dorota Duhova.

**Writing – review & editing:** Lei Fang.


