# Supplementary material for: The impact of dynamic neuromuscular stabilization on postural control and balance in post-stroke patients with sarcopenia: A case report
Source: Medicine (Baltimore). 2026 Sep 11;105(37):e50639. doi: 10.1097/MD.0000000000050639 (PMC13574404; doi:10.1097/MD.0000000000050639)
Supplement: Supplementary file 1 [file medi-105-e50639-s001.docx]

**Supplementary Table 1.** Detailed DNS Intervention Protocol with Dosage and Progression Criteria

| **Phase** | **Position** | **Training Content** | **Dosage Parameters** | **Progression Criteria** |
| --- | --- | --- | --- | --- |
| 1. Foundational Breathing & Alignment | Supine | 1. Passive postural correction (head, trunk, pelvis alignment) 2. Bilateral rib cage compression to correct rib flare 3. Diaphragmatic breathing to facilitate IAP generation | **Passive correction:** 5 min/session  **Diaphragmatic breathing:** 3 sets of 10 breaths, inhalation 4s, exhalation 6s, with 3–5s IAP hold at end-exhalation | Consistent cylindrical abdominal shape, stable IAP generation, shift from thoracic to diaphragmatic breathing pattern |
| 2. Limb Loading & Stability Training | Supine | 1. Active upper/lower limb elevation while maintaining IAP 2. Dead Bug position: alternating lower limb movements with upper limbs stable, and vice versa | **Limb elevation:** 3 sets of 10 repetitions per side IAP maintained ≥5s per repetition  **Dead Bug:** 3 sets of 10 alternating movements per side, controlled movement over 3–5s | IAP maintained ≥80% of movement time, no rib flare or compensatory trunk movement |
| 3. Positional Transition Training | Supine → Side-lying → Prone → Quadruped | 1. Controlled transitions between positions with maintained alignment 2. Quadruped anterior-posterior & medio-lateral weight shifting 3. Pelvic and lower limb dissociation drills (lateral pelvic shift with stable trunk) | **Position hold:** 3–5 min per position  **Weight shifting:** 10 repetitions per direction  **Dissociation drills:** 3 sets of 8 repetitions per side | Transitions performed without postural collapse; stable trunk during weight shifting in quadruped |
| 4. Standing Training | Standing | 1. Single-leg stance while maintaining IAP 2. Coordinated upper limb swing with stepping | **Single-leg stance:** 3 repetitions of 20–30s per side  **Stepping with arm swing:** 3 sets of 10 steps, trunk rotation ≤10° | IAP maintained during single-leg stance; minimal compensatory trunk rotation during stepping |

DNS, dynamic neuromuscular stabilization; IAP, intra-abdominal pressure
